# Differences in gonadal tissue cryopreservation practices for differences of sex development across regions in the United States

**DOI:** 10.3389/fendo.2022.990359

**Published:** 2023-01-17

**Authors:** Aisha L. Siebert, Veronica Gomez-Lobo, Emilie K. Johnson, Leena Nahata, Kyle E. Orwig, Louise C. Pyle, Selma F. Witchel, Courtney Finlayson, Monica M. Laronda

**Affiliations:** ^1^ Stanley Manne Children’s Research Institute, Ann & Robert H. Lurie Children’s Hospital of Chicago, Chicago, IL, United States; ^2^ Department of Urology, Feinberg School of Medicine, Northwestern University, Chicago, IL, United States; ^3^ Pediatric and Adolescent Gynecology, Eunice Kennedy Shriver National Institute of Child Health and Human Development, National Institutes of Health, Bethesda, MD, United States; ^4^ Division of Urology, Ann & Robert H. Lurie Children’s Hospital, Chicago, IL, United States; ^5^ Department of Pediatrics, Pediatric Endocrinology, The Ohio State University College of Medicine, Columbus, OH, United States; ^6^ Department of Obstetrics, Gynecology and Reproductive Sciences, Magee-Womens Research Institute, University of Pittsburgh School of Medicine, Pittsburgh, PA, United States; ^7^ Roberts Individualized Medical Genetics Center, Division of Human Genetics and Department of Pediatrics, Perlman School of Medicine, University of Pennsylvania, Philadelphia, PA, United States; ^8^ Division of Pediatric Endocrinology, Department of Pediatrics, University of Pittsburgh School of Medicine, Pittsburgh, PA, United States; ^9^ Division of Endocrinology, Department of Pediatrics, Ann & Robert H. Lurie Children’s Hospital, Chicago, IL, United States; ^10^ Division of Endocrinology, Department of Pediatrics, Feinberg School of Medicine, Northwestern University, Chicago, IL, United States

**Keywords:** differences of sex development (DSD), intersex, gonadal tissue cryopreservation, fertility preservation, oncofertility

## Abstract

**Objective:**

Some individuals with differences of sex development (DSD) conditions undergo medically indicated prophylactic gonadectomy. Gonads of individuals with DSD can contain germ cells and precursors and patients interested in future fertility preservation and hormonal restoration can participate in DSD-specific research protocols to cryopreserve this tissue. However, it is unclear how many providers or institutions offer gonadal tissue cryopreservation (GTC) and how widespread GTC for DSD is across the United States (US). The Pediatric Initiative Network (PIN) and Non-Oncologic Conditions committees of the Oncofertility Consortium sought to assess the current state of GTC for patients with DSD.

**Methods:**

An electronic survey was sent to providers caring for patients with DSD *via* special interest groups of professional societies and research networks.

**Results:**

The survey was administered between November 15, 2021 and March 14, 2022. A total of 155 providers responded to the survey, of which 132 respondents care for patients with DSD, and 78 work at facilities that offer medically indicated gonadectomy to patients with DSD diagnoses. They represented 55 US institutions including 47 pediatric hospitals, and 5 international sites (Canada, Denmark, Germany, Qatar). Of individual providers, 41% offer cryopreservation after prophylactic gonadectomy for patients with DSD (32/78). At an institutional level, GTC after medically indicated gonadectomy is available at 54.4% (24/46) of institutions. GTC is offered for a variety of DSD diagnoses, most commonly 45,X/46,XY DSD (i.e., Turner Syndrome with Y-chromosome material and mixed gonadal dysgenesis), ovotesticular DSD, complete androgen insensitivity syndrome (CAIS), and complete gonadal dysgenesis. Responses demonstrate regional trends in GTC practices with 83.3% of institutions in the Midwest, 66.7% in the Northeast, 54.6% in the West, and 35.3% in the South providing GTC. All represented institutions (100%) send gonadal tissue for pathological evaluation, and 22.7% preserve tissue for research purposes.

**Conclusions:**

GTC after gonadectomy is offered at half of the US institutions represented in our survey, though a minority are currently preserving tissue for research purposes. GTC is offered for several DSD conditions. Future research will focus on examining presence and quality of germ cells to support clinical decision making related to fertility preservation for patients with DSD.

## 1 Introduction

Individuals with disorders/differences of sex development (DSD) conditions, also termed intersex, experience atypical or discordant phenotype of the external genitalia in relation to the chromosomal or gonadal sex. DSD encompasses a broad range of diagnoses with variable phenotypes and malignancy risks. The indications and timing of prophylactic gonadectomy vary for individual DSD diagnoses. There is a paucity of clinical practice guidelines, but literature reviews are available on the topic of timing and indication for gonadectomy by DSD diagnosis (1, 2). Tissue removed during medically indicated gonadectomy for malignancy prevention may have future therapeutic value for fertility preservation or hormonal restoration (3).

Research on tissue maturation for future fertility or hormonal restoration is in progress, with the greatest experience coming from typical ovarian and testicular tissue. Prepubertal ovarian tissue cryopreservation (OTC) initially emerged as an experimental option for fertility preservation prior to gonadotoxic cancer therapies. In 2019 the American Society for Reproductive Medicine (ASRM) removed the experimental label because of the more than 140 reported live births following ovarian tissue transplantation of cryopreserved tissue (4–6). However, research is still ongoing, especially for prepubertal individuals undergoing OTC (7). Prepubertal testicular tissue cryopreservation (TTC) is considered experimental, but primate studies have demonstrated feasibility of grafting with return of spermatogenesis and subsequent live birth (8). Providers caring for patients with DSD may offer cryopreservation of excised gonadal tissue, or gonadal tissue cryopreservation (GTC), under a research protocol, in the hopes that current OTC and TTC maturation studies, and ongoing research into the type of cells present within gonads of different DSD diagnoses will benefit patients with DSD in the future.

Largely in response to advocacy from families and patients with DSD diagnoses, in 2018 the first US based research template for GTC in DSD was initiated (9). GTC is categorized as experimental because how the tissue may be used to offer options for biological offspring or hormone restoration has not yet been elucidated (10). It is important to note that there have been no published reports of transplantation of gonadal tissue following GTC in DSD populations, nor have any mature gametes been isolated from tissue that has been cryopreserved from these individuals. Cryopreserved gonadal tissue would most likely be used with advanced reproductive technologies (ART), and before it can be applied clinically successful demonstration of *in vitro* maturation of immature oocytes and *in vitro* spermatogenesis is necessary.

It is unclear how many providers or institutions have access to GTC for DSD, either at their own institution or through an inter-institutional agreement. To assess the current state of GTC for patients with DSD diagnoses – specifically rates of GTC after medically indicated gonadectomy for tumor prevention – as well as to inform future DSD fertility research, the Pediatric Initiative Network (PIN) and Non-Oncologic Conditions committees of the Oncofertility Consortium queried providers caring for these patients. *The aim of this survey was to assess national trends in GTC in the setting of medically indicated gonadectomy for patients with various DSD diagnoses.*

## 2 Material and methods

A notice of exemption for this study was obtained from the Ann & Robert H. Lurie Children’s Hospital of Chicago Institutional Review Board (IRB). The survey was administered to providers caring for patients with DSD between November 15, 2021 and March 14, 2022. Study data sheet and a weblink to an online Qualtrics Survey were sent out *via* the email listservs for the following organizations: American Academy of Pediatrics Special Interest Group for Clinical Geneticists; American Society for Reproductive Medicine Fertility Preservation Special Interest Group; Disorders/Differences of Sex Development – Translational Research Network; National Society of Genetic Counselors; North American Society for Pediatric and Adolescent Gynecology; Oncofertility Consortium Pediatric Initiative Network & Non-oncologic Conditions; Pediatric Endocrine Society DSD Special Interest Group; Society for Pediatric Urology; and the Society for the Study of Male Reproduction.

Participation in the study was completely voluntary, and all potential participants were provided with a study information sheet and consented to the use of response data in a descriptive summary of national trends. Potential respondents were screened, and only providers who self-identified as caring for patients with DSD were invited to complete the full survey. Respondents completed a 12-item survey developed by the multidisciplinary research team, consisting of multiple-choice and free text responses enquiring about gonadal care, including gonadectomy and GTC practices by individual DSD diagnoses (**Supplemental Table 1**). The online survey took approximately five minutes to complete.

## 3 Results

### 3.1 Many individual providers and institutions offer GTC for DSD diagnoses

A total of 155 providers initiated the survey and 132 respondents indicated that they care for patients with DSD. Because lists of subscribers to the listservs used to distribute the survey were not shared, the response rate could not be calculated. Fifty-two responses were excluded because they did not provide an email address or institution and therefore could not be verified as a unique responder. Respondents represented 55 US institutions including 47 pediatric hospitals, and 5 international sites with two facilities in Canada, and one each in Denmark, Germany, and Qatar. The majority of responses were from US based providers with up to five responses from a single institution.

Of respondents who completed the full survey, 97.5% (78/80) individual providers offer medically indicated gonadectomy for patients with DSD, of which 41% (32/78) offer cryopreservation of excised gonadal tissue. GTC is available at 54.4% (25/46) institutions represented in the survey that reported on availability of this procedure. Only 9 providers indicated age at which cryopreservation is offered, collectively indicating a range of 0 to 100 years, with the average minimum age of 5.7 years and average maximum age of 25.8 years. Practice patterns of multiple providers at the same institution demonstrated a high degree of concordance in survey responses, with only two institutions in which one provider indicated “Don’t know” and another provided diagnosis and/or age range information. In such cases, the more complete response was used for that institution

### 3.2 Institutional practice patterns were assessed by US census region

**Figure 1:**
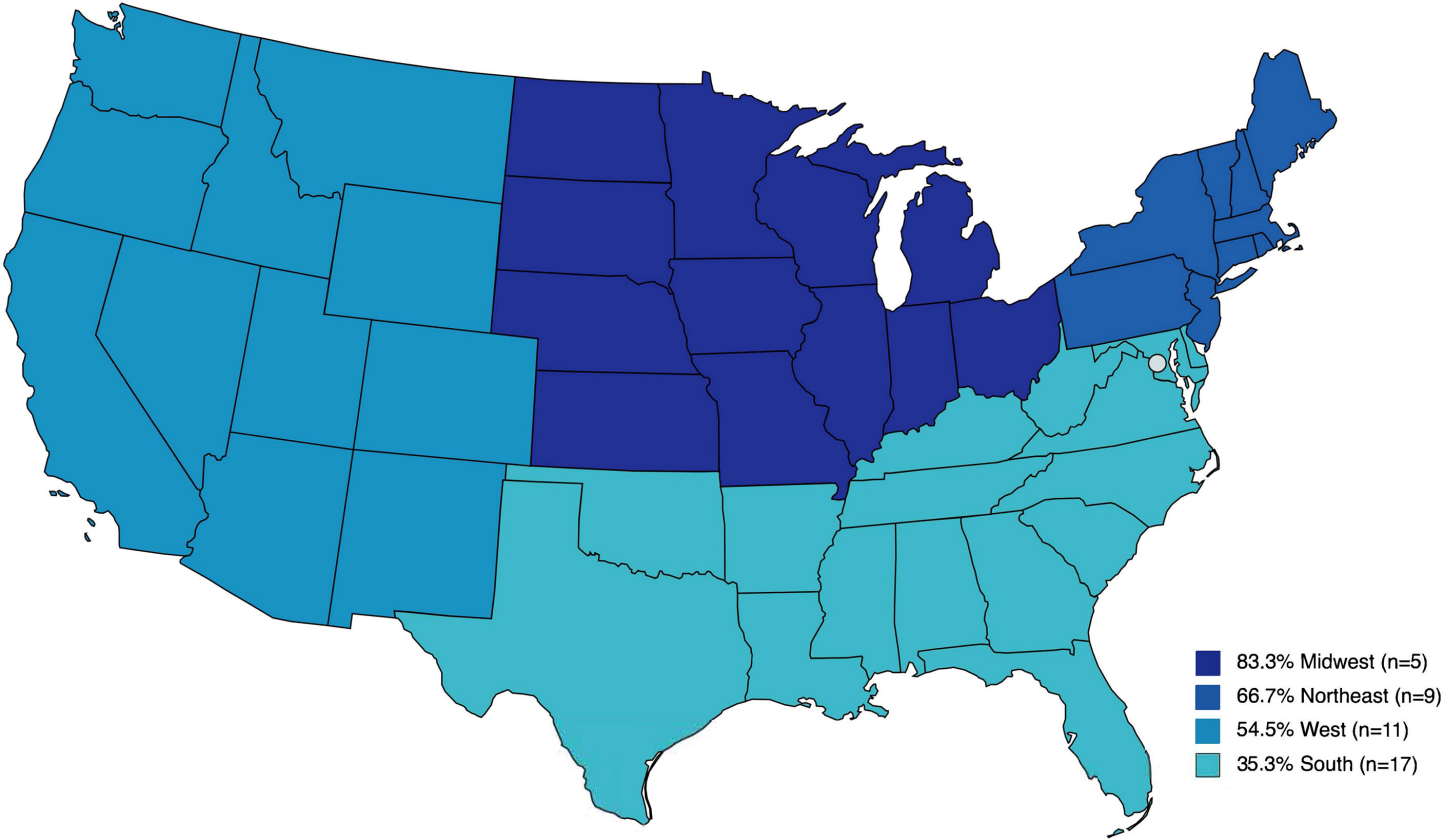
Percent of institutions that participated in the survey and offer gonadal tissue cryopreservation by United States census region.

**Figure 2:**
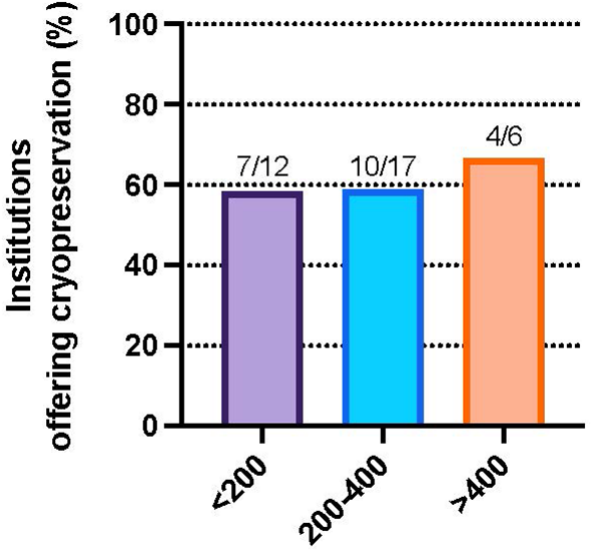
Percent of institutions that participated in the survey and offer gonadal cryopreservation for DSD patients by facility beds, <200 beds (58.3%), 200-400 beds (58.8%), and >400 beds (66.7%), p=NS.

To evaluate regional differences, responses were aggregated by institution and analysis was performed on the US subset of represented institutions. To assess trends by geographic location, the proportion of institutions offering GTC was examined across US census regions. The highest percentage of facilities offering GTC were from the Midwest 83.3% (5/6), followed by 66.7% (6/9) in the Northeast, 54.6% (6/11) in the West and 35.3% (6/17) in the South (**Figure 1**). Although the observed differences were not statistically significant (Fisher exact test, p=0.77), we did note that the regions with the largest number of institutions represented in our survey were the least likely to offer cryopreservation. We examined institute or facility size as a possible confounder of the observed regional differences. The proportion of institutions offering GTC were not different by number of hospital beds (Fisher exact test, p>0.99) (**Figure 2**).

**Figure 3:**
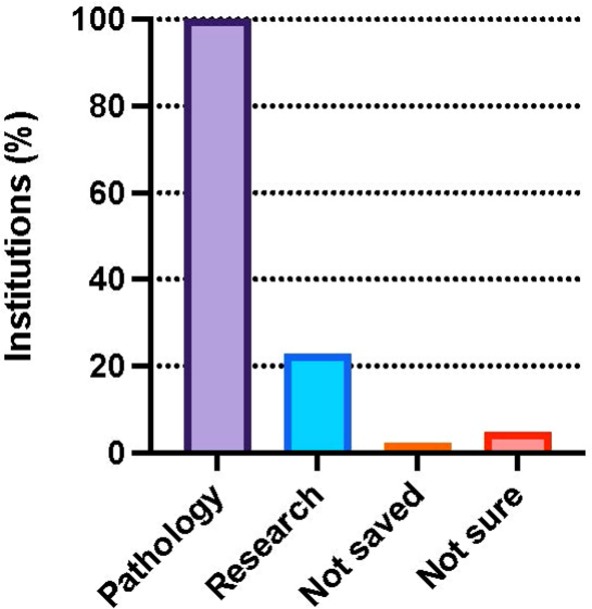
Percent of institutions that participated in the survey and perform pathology (100.0%) and research (22.7%) on tissue isolated from DSD patients. Few institutions reported tissue is not saved (2.3%) or were unsure (4.5%). Survey participants could choose more than one response.

One hundred percent (44/44) of the institutions that report offering gonadectomy, and which provided information on tissue applications send gonadal tissue for pathological evaluation. Only 22.7% (10/44) of institutions preserve tissue for research purposes, including work to advance future therapeutic application of cryopreserved tissue (**Figure 3**). Of institutions offering GTC and reporting on where tissue it cryopreserved, 55% (11/20) perform tissue freezing on site and the remaining 45% (9/20) ship samples to another center for cryopreservation.

### 3.3 GTC is offered for all surveyed DSD diagnoses

**Figure 4:**
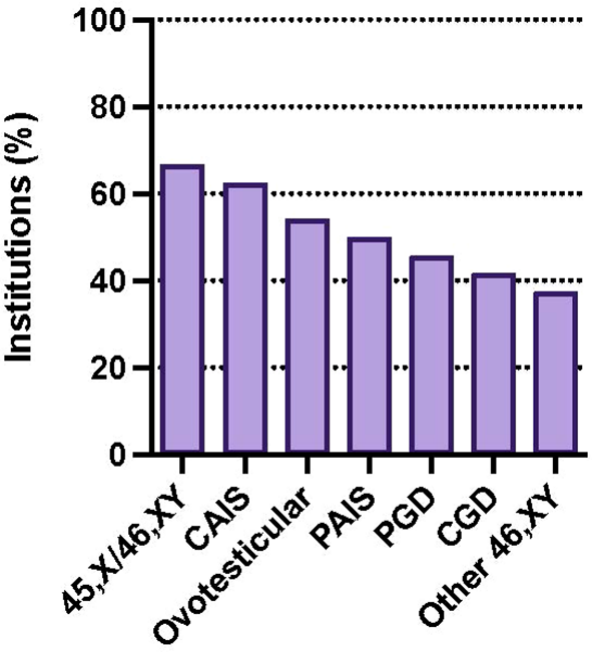
Percentage of institutions offering GTC by diagnosis: 45,X/46,XY DSD (66.7%), CAIS (62.5%), ovotesticular DSD (54.2%), PAIS (50%), partial gonadal dysgenesis (PGD) (45.8%), complete gonadal dysgenesis (CGD) (41.7%). Survey participants could choose more than one response.

GTC is offered at participating institutions for a variety of DSD diagnoses following medically indicated gonadectomy, most commonly 45,X/46,XY DSD (66.7%), followed by CAIS (62.5%), Ovotesticular DSD (54.2%), and PAIS (50%). Patients with partial (45.8%) and complete gonadal dysgenesis (41.7%) are least likely to be offered cryopreservation (**Figure 4**).

## 4 Discussion

Our survey showed over a third of DSD providers who work at institutions that perform gonadectomy for DSD report offering GTC for this population. Gonadectomy was most commonly offered for conditions with the greatest malignancy risk including 45,X/46,XY DSD, complete and partial gonadal dysgenesis, and PAIS. GTC was offered most commonly for 45,X/46,XY DSD, CAIS, ovotesticular DSD, and PAIS. Respondents are least likely to offer cryopreservation after gonadectomy for partial and complete gonadal dysgenesis.

Understanding the current state of GTC for DSD is important because prior research indicates that future fertility is a concern for patients with DSD, who are open to many family-building options and desire autonomy in their decision making (11, 12). To be responsive to patient and family needs, a GTC for DSD research protocol has been developed that shows that pathologic evaluation of half of the gonad can be safely used to assess tumor burden and borders as well as presence of germ cells following medically indicated gonadectomy. If no tumor is present, the remaining half of the gonad can be cryopreserved for long term storage and potential future fertility or hormone restoration (9). Individual patients may also elect to donate a small portion of tissue to ongoing research. Though this concept was published from one institution, the present study indicates that GTC for DSD is being operationalized at 25 institutions in the US while less than a third of those engaging in research to advance clinical applications of that tissue.

A retrospective study of gonadal biopsy specimens from patients with DSD showed germ cells in some gonads from patients who previously would have been assumed to be infertile (e.g., streak ovaries, dysgenetic gonads, ovotestes), particular for the youngest patients aged 0 to 3 years old. (13). Additional research that identifies the type of germ cells present, their point in mitosis or meiosis, the type of supportive somatic cells and the quality of resulting gametes must be performed to assess the restorative potential of these germ cells. This research will add to the personalized care of this heterogenous patient populations that fall under the umbrella term of DSD, and may provide further understanding of the mechanisms of gonadal sex development. Through our recent survey, we now have a better appreciation for the scope of GTC availability for DSD, which will facilitate future collaborative studies.

We mapped the number of institutions that offered GTC across the US. In our survey, participating institutions in the Midwest were most likely, whereas institutions in the South were least likely to offer GTC. Individual provider and institutional responses that care for DSD patients spanned the continental US and there was no difference in the size of the facility that indicated the likelihood of a facility to offer GTC. There is a paucity of information assessing future fertility potential of cryopreserved gonadal tissue for individuals with DSD. Practice responses in our survey span all size institutions and almost half of represented institutions that offer GTC utilize an inter-institutional agreement for this service. There is a great opportunity for research on a larger scale through inter-institutional collaborations to inform future clinical care for DSD diagnoses.

### 4.1 Limitations

Our population lacks a denominator estimate, therefore making it impossible to assess response rate. We surveyed membership of 12 different organizations to which providers caring for patients with DSD may belong and we cannot determine the degree of overlap between these large listservs, the number of their membership who care for patients with DSD diagnoses, nor the number of providers who care for individuals with DSD but do not belong to these professional societies or subscribe to their listservs. Additionally, the number of providers or institutions who offer GTC are based on respondent reports and were not independently verified by chart review. Respondents provided aggregate reporting of cryopreservation practices by diagnosis as well as limited information on age at which gonadal care services are performed for various DSD diagnoses. Observed trends may reflect either differences in practice patterns, sampling bias, or a combination of the two. Cost of GTC varies by circumstance and insurance and may significantly impact access. In our experience the average cost of GTC is $1000 per patient when surgical cost is bundled with other oncologic care (14). Additional follow-up studies are needed to understand indications and timing of GTC following medically indicated gonadectomy for patients with various DSD diagnoses in the United States.

## Data availability statement

The original contributions presented in the study are included in the article/**Supplementary Material**. Further inquiries can be directed to the corresponding author.

## Author contributions

AS adapted the survey to an online format, compiled responses, analysed data, and prepared results for publication. EJ, CF, and ML developed the survey content. AS, EJ, VG-L, LN, KO, LP, SW, CF, and ML distributed the survey *via* professional organization listservs and provided critical input on survey data interpretation. All authors contributed to the article and approved the submitted version.

## Supplementary material

Supplementary Table 1 Gonadal Care Survey text
