# Supplementary material for: Differences in gonadal tissue cryopreservation practices for differences of sex development across regions in the United States
Source: Front Endocrinol (Lausanne). 2023 Jan 17;13:990359. doi: 10.3389/fendo.2022.990359 (PMC9886870; doi:10.3389/fendo.2022.990359)
Supplement: Supplementary Table 1 — Gonadal Care Survey text [file Table_1.docx]

| **Number** | **Question** | **Response Options** |
| --- | --- | --- |
| Screening Question. | Do you care for patients with differences of sex development (DSD)? | Yes, No |
| Q1. | Yes: Thank you for agreeing to take part in this survey. The survey is intended to gather  information on current practice patterns among providers caring for patients with differences of  sex development (DSD). Participation is completely voluntary. By filling out this survey you  agree to the use of your responses in a descriptive summary of national trends. Individual  institutions and study participants will not be identified in any public presentation of these data.  Study staff may contact you regarding future participation in experimental protocols for gonadal  tissue cryopreservation for patients with DSD. | No: Thank you for responding to this online survey. The survey is intended to gather information  on current practice patterns among providers caring for patients with differences of sex  development (DSD). |
|  | Please enter your email address: | Free text |
| Q2. | Name of institution where you provide care: | Free text |
| Q3. | Do surgeons at your institution perform gonadectomy for patients with differences of sex  development (DSD) diagnoses? | - Yes (go to Q4)  - No (skip to Q12). |
| Q4. | For which DSD conditions do you offer gonadectomy? | Check all that apply:  - 45,X/46,XY conditions with elevated malignancy potential, including Turner Syndrome  and Mixed Gonadal Dysgenesis  - Ovotesticular DSD  - Complete Gonadal Dysgenesis  - Partial Gonadal Dysgenesis  - Complete Androgen Insensitivity Syndrome  - Partial Androgen Insensitivity Syndrome  - Other 46,XY conditions associated with impaired androgen production (ex. 5-alpha  reductase deficiency, 17-beta HSD3  deficiency)_________________________________(write in)  - Not sure |
| Q5. | What is the age range for gonadectomy patients at your institution? Please give your best  estimate. | - Youngest (years)  - Oldest (years) |
| Q6. | How is gonadal tissue saved at your institution? Check all that apply: | - For pathological evaluation  - For research  - For some other reason_______________________________________-  - Gonadal tissue is not saved  - Not sure |
| Q7. | Do you offer gonadal tissue cryopreservation* to patients with DSD?  (*Offer tissue freezing for DSD patients in hopes that the tissue may be able to be used to  produce a biological child using assisted reproductive technology in the future) | - Yes (go to Q8)  - No (Skip to Q12) |
| Q8. | Which of the following DSD diagnoses do you offer gonadal tissue cryopreservation for? | Check all that apply:  - 45,X/46,XY conditions with elevated malignancy potential, including Turner Syndrome  and Mixed Gonadal Dysgenesis  - Ovotesticular DSD  - Complete Gonadal Dysgenesis  - Partial Gonadal Dysgenesis  - Complete Androgen Insensitivity Syndrome  - Partial Androgen Insensitivity Syndrome  - Other 46,XY conditions associated with impaired androgen production (ex. 5-alpha  reductase deficiency, 17-beta HSD3 deficiency)  - Not sure  - None |
| Q9. | Is it performed at your institution or external site? | Check all that apply:  - My institution  - Other institution (write in name of institution)______________________  - Not sure |
| Q10. | Do you perform gonadal tissue cryopreservation for a DSD diagnosis under an IRB  research protocol? | - Yes (Go to Q11)  - No (skip to Q12) |
| Q11. | What age range is included in your protocol? | - Youngest (years)  - Oldest (years) |
| Q12. | Use this space to provide any additional comments about your DSD program, particularly  related gonadectomy or gonadal tissue cryopreservation: | Free text |
| We thank you for your time spent taking this survey.  Your response has been recorded. | | |
